# Reduction of bias in the evaluation of fractional anisotropy and mean diffusivity in magnetic resonance diffusion tensor imaging using region-of-interest methodology

**DOI:** 10.1038/s41598-019-49311-w

**Published:** 2019-09-11

**Authors:** Youngseob Seo, Nancy K. Rollins, Zhiyue J. Wang

**Affiliations:** 1 Center for Medical Metrology, Korea Research Institute of Standards and Science, Daejeon, Republic of Korea; 2 Department of Radiology, University of Texas Southwestern Medical Center, Dallas, TX USA; 3 Department of Radiology, Children’s Health, Dallas, TX USA

**Keywords:** Brain imaging, Magnetic resonance imaging, Diagnostic markers, Predictive markers

## Abstract

Accurate quantification of fractional anisotropy (FA) and mean diffusivity (MD) in MR diffusion tensor imaging (DTI) requires adequate signal-to-noise ratio (SNR) especially in low FA areas of the brain, which necessitates clinically impractical long image acquisition times. We explored a SNR enhancement strategy using region-of-interest (ROI)-based diffusion tensor for quantification. DTI scans from a healthy male were acquired 15 times and combined into sets with different number of signal averages (NSA = 1–4, 15) at one 1.5-T Philips and three 3-T (Philips, Siemens and GE) scanners. Equivalence test was performed to determine NSA thresholds for bias-free FA and MD quantifications by comparison with reference values derived from images with NSA = 15. We examined brain areas with low FA values including caudate nucleus, globus pallidus, putamen, superior temporal gyrus, and substructures within thalamus (lateral dorsal, ventral anterior and posterior nuclei), where bias-free FA is difficult to obtain using a conventional approach. Our results showed that bias-free FA can be obtained with NSA = 2 or 3 in some cases using ROI-based analysis. ROI-based analysis allows reliable FA and MD quantifications in various brain structures previously difficult to study with clinically feasible data acquisition schemes.

## Introduction

Magnetic resonance diffusion tensor imaging (DTI) provides information about water diffusion with the dominant direction parallel to the axonal orientation within the voxel of interest^1–3^. DTI enables the assessment of white and gray matter integrity in normal development and many disease states, by providing quantitative measures of fractional anisotropy (FA) and diffusivities, especially mean diffusivity (MD)^4–9^, that are biomarkers. DTI is usually acquired with phased array coils using parallel imaging methods to reduce image acquisition (ACQ) time and head motion artifacts. Using phased array coils, the signal-to-noise (SNR) within the acquired images is inhomogeneous with higher SNR peripherally and lower SNR centrally in the brain^10–12^. The reproducibility, precision and accuracy of estimating tensor metrics are affected by SNR and analytic methodologies, such as operator-dependent manual region-of-interest (ROI) analysis for a single subject DTI data and operator-independent tract-based spatial statistics (TBSS)^13^ for analyzing multi-subject tensor data.

In the conventional data processing approaches, FA and MD values are calculated for each voxel, and maps are generated using whole brain automated analysis. The results are further assessed over ROIs defined by the operator. Bias-free measurements of FA require adequate SNR and regions with inherently lower FA require higher SNR^14–16^. For regions of white matter which have high degrees of anisotropy such as genu and splenium of the corpus callosum, bias-free determination of FA is relatively easy to achieve. In a previous work^14^, on the other hand, it has been determined that bias free measurement of FA in the low FA region (putamen) at 1.5 T and 3 T requires at least number of signal average (NSA) = 9 and 6, respectively. In our institutes, three DTI scans are the maximum because patients also undergo other MRI scans. The diffusion scans are limited to less than 20 minutes. Diffusion data with NSA = 9 requires almost one hour to complete whole brain coverage, which is considered to be too long, and may not be practical for motion free scans in many study participants.

DTI of low FA regions of the brain is a challenge because of magnetic field strength limitation of MRI scanners and examination time restrictions of clinical examinations. In this study, we investigated an ROI-based tensor processing method in which image intensities inside an ROI with uniform diffusion properties are averaged first before calculating the diffusion tensor^17,18^. This method mitigates the requirement of long image acquisition times needed for bias-free FA and MD measurements and has not been applied to brain studies previously.

## Results

### Visual inspection of motion-corrupted diffusion data and calculation of ROI size

Data acquired from all vendors did not contain image volumes with motion-induced image artifacts or missing slices irrespective to a long period of scan times. Table 1 shows both number of voxels in the selected ROIs and ROI sizes including sub-ROIs.

**Table 1:** Number of reconstructed image voxels and size in the selected ROIs.

Brain region	Number of voxels in ROI (mean ± SEM)	ROI size [mm^3^] (mean ± SEM)	Sub-ROI*	Number of voxels in sub-ROI (mean ± SEM)	Sub-ROI size [mm^3^] (mean ± SEM)
CN	89 ± 6	172.5 ± 10.8	1	22 ± 3	43.6 ± 5.4
			2	22 ± 2	44.2 ± 4.3
			3	22 ± 3	43.2 ± 5.8
			4	23 ± 3	47.1 ± 5.9
GP	98 ± 7	190.8 ± 13.4	1	26 ± 4	51.6 ± 7.6
			2	24 ± 3	47.1 ± 6.1
			3	25 ± 4	51.3 ± 7.5
			4	23 ± 3	45.8 ± 5.2
PUT	122 ± 7	236.4 ± 12.7	1	29 ± 3	57.8 ± 5.6
			2	31 ± 4	60.8 ± 6.7
			3	32 ± 4	62.7 ± 6.9
			4	30 ± 3	58.6 ± 6.1
STG	140 ± 8	271.2 ± 15.0	1	34 ± 3	67.2 ± 7.3
			2	35 ± 4	68.1 ± 7.5
			3	35 ± 4	68.8 ± 7.4
			4	36 ± 4	72.1 ± 7.7
Thalamus_LD	78 ± 5	151.3 ± 9.4	1	19 ± 3	38.4 ± 4.5
			2	19 ± 2	37.4 ± 4.2
			3	19 ± 2	39.1 ± 3.6
			4	21 ± 3	41.4 ± 4.7
Thalamus_VA	62 ± 5	120.6 ± 8.2	1	17 ± 3	33.2 ± 4.3
			2	15 ± 2	28.4 ± 4.2
			3	14 ± 2	27.5 ± 3.4
			4	16 ± 3	31.5 ± 4.1
Thalamus_VP	69 ± 6	134.8 ± 8.6	1	17 ± 3	32.8 ± 4.8
			2	17 ± 3	33.3 ± 5.2
			3	18 ± 3	34.9 ± 4.9
			4	17 ± 3	33.8 ± 5.1

CN, caudate nucleus; GP, globus pallidus; PUT, putamen; SEM, standard error of the mean; STG, superior temporal gyru; LD, lateral dorsal; VA, ventral anterior; VP, ventral posterior. *Sub-ROIs 1 and 2 are two sub-ROIs on Slice 1; Sub-ROIs 3 and 4 are two sub-ROIs on Slice 2.

### SNR and Intra-ROI diffusion direction dispersion angle (IRDDDA)

**Figure 1:**
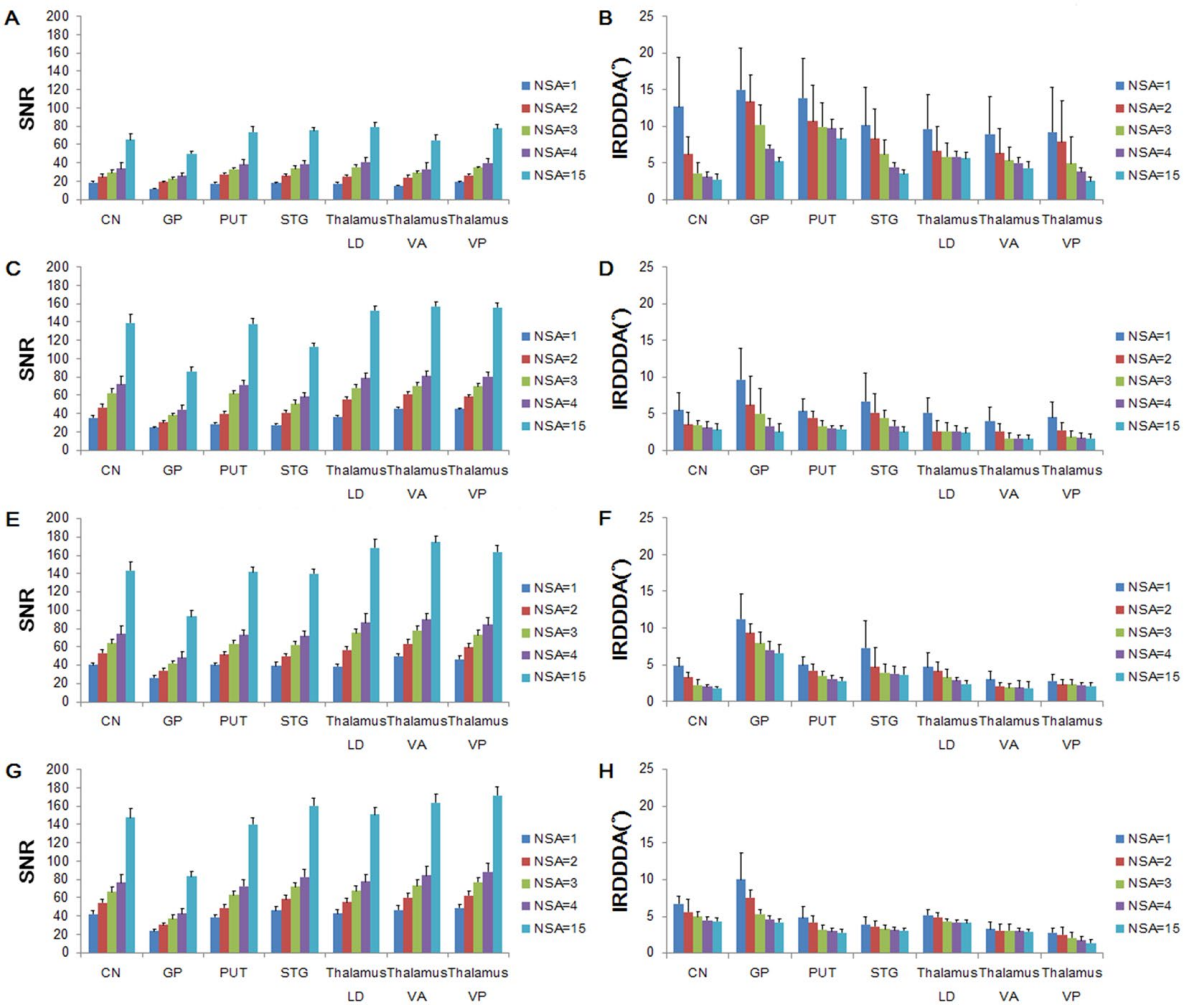
Signal-to-noise ratio (SNR) and intra-ROI diffusion direction dispersion angle (IRDDDA) of brain ROIs for different number of signal averages (NSA) at 1.5 T Philips (**A**,**B**), 3 T Philips (**C**,**D**), 3 T Siemens (**E**,**F**) and 3 T GE (**G**,**H**) systems. T bars represent standard deviations. Abbreviations: CN, caudate nucleus; GP, globus pallidus; LD, lateral dorsal; NSA, number of signal average; PUT, putamen; ROI, region-of-interest; SNR, signal-to-noise ratio; STG, superior temporal gyrus; VA, ventral anterior; VP, ventral posterior.

**Figure 2:**
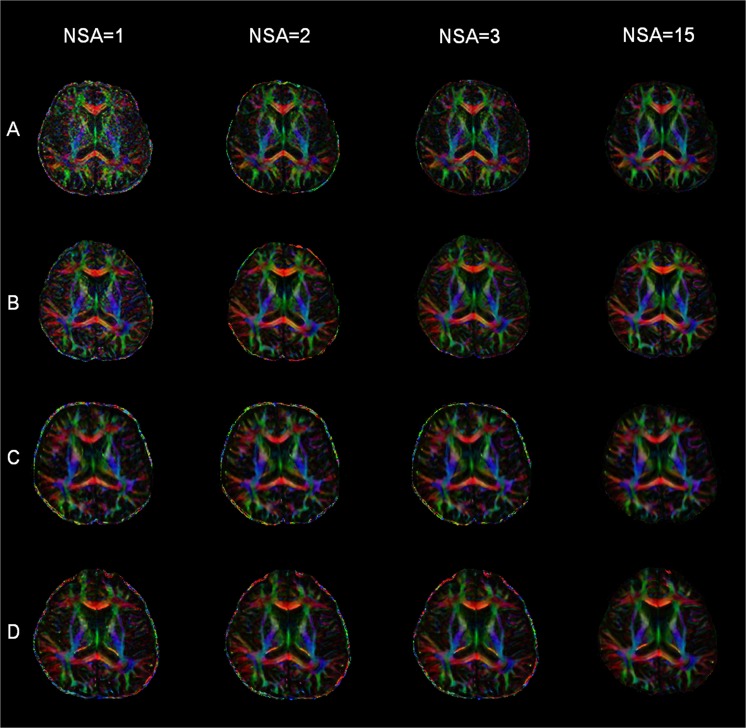
Color-coded FA images with NSA = 1, 2, 3 and 15 are displayed for 1.5 T Philips (**A**), 3 T Philips (**B**), 3 T Siemens (**C**) and 3 T GE (**D**) scanners.

Both SNR and IRDDDA values were estimated in the selected regions of the brain in terms of NSA for four different MRI scanners (Fig. 1). The SNR varied across the brain depending on the MRI scanners and was proportional to the square root of NSA. The IRDDDA decreased as the NSA increased across the brain ROIs. Higher SNR and smaller IRDDDA values at 3 T were obtained than those at 1.5 T. Figure 2 shows FA images with transversal slice orientation of the brain processed data with NSA of 1, 2, 3 and 15 on four different MRI scanners. The effect of SNR is apparent on these FA maps. Supplement Tables 1–4 show the percent differences in SNR between ACQ group (1, 2 and 3) at the beginning of the MRI session and the subsequent ACQ group (13, 14 and 15) in terms of the selected regions of the brain on the MRI scanners. SNR values derived from earlier and later acquisitions differed by ≤8.3%.

### Voxel-based and ROI-based mean FA and MD values derived from images with NSA = 15

Mean FA and MD values of the reference DTI with NSA = 15 in the selected regions of the brain on different MRI scanners are shown in Table 2. The voxel-based mean FA and MD values were used as standard reference values for subsequent equivalence testing with 90% confidence intervals (CIs) for mean FA and MD measurements to determine the minimum NSA for bias-free FA and MD in the selected regions of the brain. The ROI-based values agree well with the voxel-based ones in most regions.

**Table 2:** ROI-based and voxel-based FA and MD values (mean (SD)) from images with NSA = 15 at four different MRI systems.

Location			CN	GP	PUT	STG	Thalamus LD	Thalamus VA	Thalamus VP
Fractional Anisotropy	1.5 T Philips	ROI-based	0.20 (0.01)	0.21 (0.01)	0.19 (0.01)	0.36 (0.02)	0.23 (0.02)	0.33 (0.02)	0.33 (0.01)
		Voxel-based	0.22 (0.01)	0.24 (0.02)	0.22 (0.02)	0.39 (0.02)	0.25 (0.02)	0.35 (0.03)	0.34 (0.02)
	3 T Philips	ROI-based	0.19 (0.01)	0.23 (0.01)	0.19 (0.01)	0.40 (0.02)	0.22 (0.01)	0.31 (0.01)	0.39 (0.02)
		Voxel-based	0.20 (0.01)	0.26 (0.01)	0.21 (0.01)	0.42 (0.03)	0.23 (0.02)	0.33 (0.02)	0.40 (0.03)
	3 T Siemens	ROI-based	0.21 (0.01)	0.22 (0.01)	0.19 (0.01)	0.34 (0.02)	0.21 (0.01)	0.33 (0.01)	0.38 (0.01)
		Voxel-based	0.23 (0.01)	0.26 (0.02)	0.21 (0.02)	0.36 (0.03)	0.23 (0.01)	0.35 (0.02)	0.39 (0.02)
	3 T GE	ROI-based	0.18 (0.01)	0.21 (0.01)	0.20 (0.01)	0.33 (0.02)	0.22 (0.01)	0.33 (0.02)	0.36 (0.02)
		Voxel-based	0.20 (0.01)	0.24 (0.02)	0.22 (0.02)	0.34 (0.02)	0.23 (0.01)	0.35 (0.02)	0.39 (0.02)
Mean Diffusivity (10^−3^ mm^2^/s)	1.5 T Philips	ROI-based	0.68 (0.02)	0.67 (0.03)	0.71 (0.02)	0.73 (0.02)	0.75 (0.02)	0.70 (0.01)	0.74 (0.01)
		Voxel-based	0.70 (0.03)	0.70 (0.03)	0.73 (0.04)	0.75 (0.02)	0.79 (0.03)	0.74 (0.03)	0.76 (0.03)
	3 T Philips	ROI-based	0.65 (0.02)	0.62 (0.03)	0.67 (0.02)	0.69 (0.02)	0.73 (0.02)	0.69 (0.02)	0.67 (0.02)
		Voxel-based	0.66 (0.03)	0.64 (0.03)	0.69 (0.03)	0.71 (0.03)	0.76 (0.04)	0.70 (0.02)	0.70 (0.02)
	3 T Siemens	ROI-based	0.66 (0.03)	0.65 (0.02)	0.72 (0.03)	0.75 (0.03)	0.76 (0.02)	0.71 (0.02)	0.70 (0.02)
		Voxel-based	0.67 (0.03)	0.66 (0.03)	0.73 (0.03)	0.76 (0.03)	0.79 (0.03)	0.73 (0.03)	0.73 (0.02)
	3 T GE	ROI-based	0.69 (0.02)	0.66 (0.02)	0.69 (0.02)	0.69 (0.02)	0.72 (0.02)	0.76 (0.02)	0.71 (0.02)
		Voxel-based	0.71 (0.03)	0.68 (0.03)	0.70 (0.03)	0.72 (0.03)	0.74 (0.03)	0.79 (0.03)	0.74 (0.02)

CN, caudate nucleus; FA, fractional anisotropy; GP, globus pallidus; LD, lateral dorsal; MD, mean diffusivity; NSA, number of signal average; PUT, putamen; ROI, region-of-interest; SD, standard deviation; STG, superior temporal gyrus; VA, ventral anterior; VP, ventral posterior.

### Equivalence testing of DTI metrics relative to reference values for images with different NSAs

**Figure 3:**
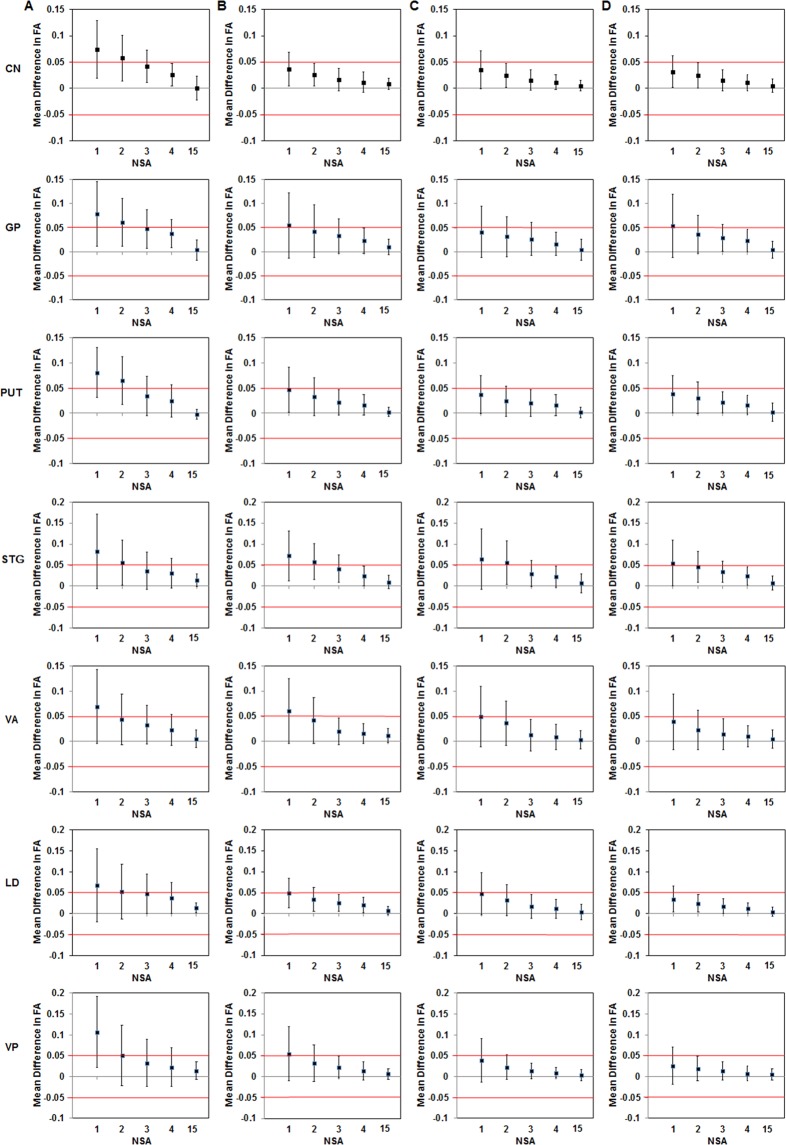
Equivalence testing for the determination of the minimum NSA for bias-free FA evaluation is shown for ROI-based analysis at 1.5 T Philips (**A**), 3 T Philips (**B**), 3 T Siemens (**C**) and 3 T GE (**D**) MRI systems (Equivalence tolerance range = [−0.05, 0.05]). FA, fractional anisotropy; NSA, number of signal average.

The 90% CIs of error relative to the reference values for both ROI- and voxel-based FA values in terms of number of acquisitions are shown in Fig. 3 and Supplementary Fig. 1. ROI-based FA values with NSA = 15 were equivalent to the reference values(=voxel-based FA values with NSA = 15) in the selected regions for four different MRI systems. Suggested minimum number of acquisitions needed for bias-free FA and MD measurements using both ROI- and voxel-based quantifications is summarized in Table 3.

**Table 3:** Suggested minimum number of acquisitions needed for bias-free FA and MD measurements using both ROI- and voxel-based quantifications at 1.5 T and 3 T MRI scanners (voxel-based values are in parenthesis).

	Brain region	1.5 T Philips	3 T Philips	3 T Siemens	3 T GE
Fractional Anisotropy	CN	4 (N/A)	2 (3)	2 (3)	2 (3)
	GP	N/A (N/A)	4 (N/A)	4 (N/A)	4 (N/A)
	PUT	N/A (N/A)	3 (N/A)	3 (N/A)	3 (N/A)
	STG	N/A (N/A)	4 (N/A)	4 (N/A)	4 (N/A)
	Thalamus VA	N/A (N/A)	3 (N/A)	3 (4)	3 (4)
	Thalamus LD	N/A (N/A)	3 (4)	3 (4)	3 (4)
	Thalamus VP	N/A (N/A)	3 (4)	3 (4)	2 (4)
Mean Diffusivity	CN	N/A (N/A)	N/A (N/A)	N/A (N/A)	N/A (N/A)
	GP	N/A (N/A)	N/A (N/A)	N/A (N/A)	N/A (N/A)
	PUT	4 (N/A)	3 (4)	3 (4)	3 (4)
	STG	N/A (N/A)	3 (4)	3 (4)	3 (4)
	Thalamus VA	N/A (N/A)	3 (N/A)	3 (N/A)	3 (N/A)
	Thalamus LD	N/A (N/A)	N/A (N/A)	N/A (N/A)	N/A (N/A)
	Thalamus VP	3 (4)	2 (3)	2 (3)	2 (3)

ACQ, acquisition; CN, caudate nucleus; FA, fractional anisotropy; GP, globus pallidus; LD, lateral dorsal; MD, mean diffusivity; NSA, number of signal average; PUT, putamen; SNR, signal-to-noise ratio; STG, superior temporal gyrus; VA, ventral anterior; VP, ventral posterior. N/A = not applicable due to minimum number of acquisitions >4.

For ROI-based FA at 1.5 T MRI scanner, CIs of error relative to reference values were not within the equivalence tolerance range of [−0.05, 0.05] for the selected regions of the brain at NSA ≤ 3, but the 90% CI in the CN only was within the equivalence tolerance at NSA = 4 (Fig. 3A), while for voxel-based FA at 1.5 T, CIs were not within the equivalence tolerance range for all the selected regions (Supplementary Fig. 1A).

For ROI-based FA at 3 T, all the selected regions of the brain except GP and STG had equivalence threshold at NSA = 2 or 3, and all the selected regions had equivalence threshold at NSA = 4 (Fig. 3B–D). For voxel-based FA at 3 T, CN only had equivalence threshold at NSA = 3, and CN, VA, LD and VP had equivalence threshold at NSA = 4 (Supplementary Fig. 1B–D).

**Figure 4:**
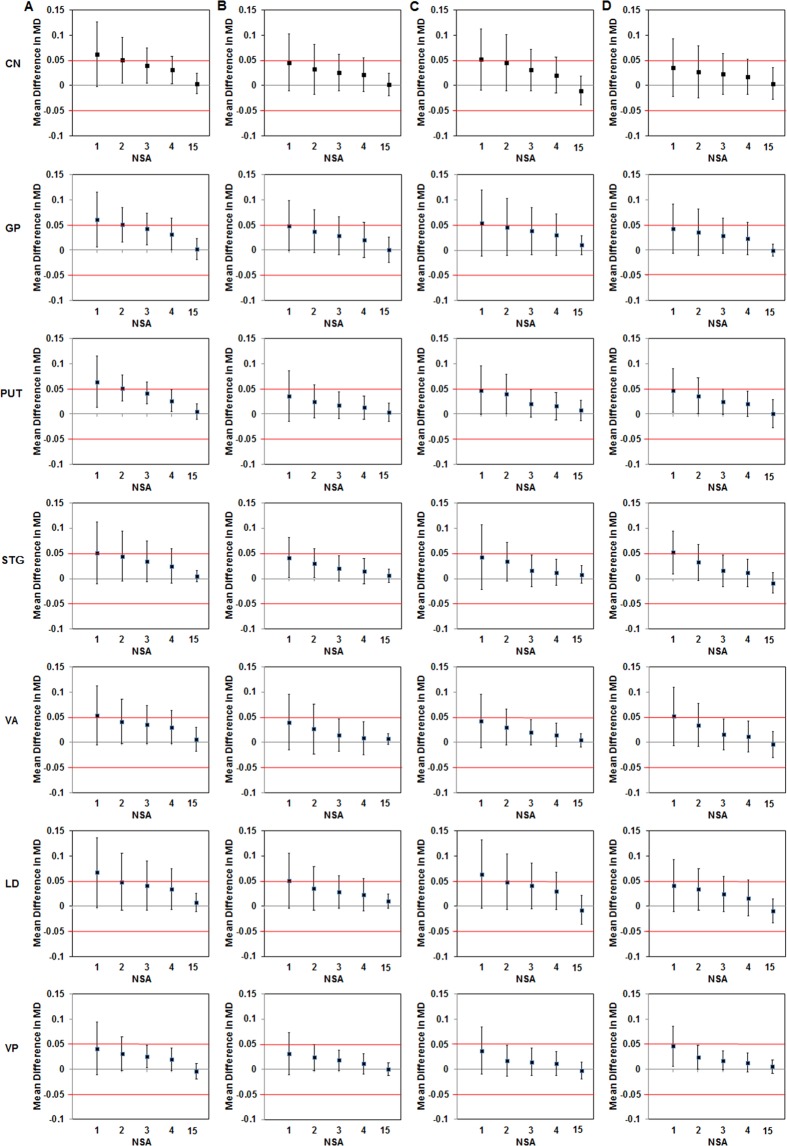
Equivalence testing for the determination of the minimum NSA for bias-free MD evaluation is shown for ROI-based analysis at 1.5 T Philips (**A**), 3 T Philips (**B**), 3 T Siemens (**C**) and 3 T GE (**D**) MRI systems (Equivalence tolerance range = [−0.05, 0.05]). MD, mean diffusivity; NSA, number of signal average.

The 90% CIs of error relative to the reference values for both ROI- and voxel-based MD in terms of NSA are shown in Fig. 4 and Supplementary Fig. 2. For ROI-based MD at 1.5 T, VP only had equivalence threshold at NSA ≥ 3 and PUT at NSA = 4 (Fig. 4A). For ROI-based MD at 3 T, PUT, STG, VA and VP had equivalence threshold at NSA ≥ 2 or 3 (Fig. 4B–D).

For voxel-based MD at 1.5 T, CIs of error were not within the equivalence tolerance range of [−0.05, 0.05] for all the selected regions of the brain except VP (Supplementary Fig. 2A), while, for voxel-based MD at 3 T, PUT and STG had equivalence threshold at NSA = 4 and VP at NSA ≥ 3 (Supplementary Fig. 2B–D).

## Discussion and Conclusion

Inadequate SNR leads to overestimation of the largest and underestimation of the smallest eigenvalues respectively^14,19,20^. The resultant technical bias in FA can mask or mimic disease processes^15,21^. For lower SNR, this problem is more pronounced in low FA areas of the brain than high FA regions^14^. A previous study demonstrated that with NSA ≥ 9 at 1.5 T, the measurements of FA for the putamen were bias-free^14^. With the high SNR provided by 9 and 6 NSAs, reproducible FA values for the putamen were seen at 1.5 T and 3 T, respectively.

While the motion is likely to affect SNR during a long period of scan time, our diffusion data obtained from all MRI scanners show that SNR of early acquisitions are similar with that acquired later. These results suggest that the head motion and other factors (gradient warming etc.) did not affect SNR for 15 tensor data sets that were obtained.

In this study, the ROI-based FA values were slightly lower than those derived from voxel-based analysis, likely caused by the variability of the primary direction of the diffusions within the ROI. The ROI was drawn on a color-coded FA map to ensure that voxels within the ROI were relatively uniform. The voxel-based FA was biased, and the bias was greater when the NSA was lower. The bias for the ROI-based FA values was much less sensitive to SNR than voxel-based values. FA measurement derived from 1.5 T diffusion data with NSA ≤ 3 in the low FA regions cannot be used for disease or response to therapy in the brain as an imaging biomarker because low SNR in these regions lead to a significant bias in tensor metrics. However, in the CN, PUT, thalamus VA, LD and VP, the error of FA measurement derived from our proposed method at 3 T is relatively low at NSA = 2 or 3 as compared to reference values.

Furthermore, the bias for MD measurements using ROI-based analysis in low FA regions also was smaller than that with voxel-based analysis, and the errors were smaller for the ROI-based method. In the thalamus VP particularly, the bias for ROI-based MD values was relatively small as compared with that for ROI-based FA at NSA = 2 or 3 suggesting that MD measurement derived from ROI-based analysis at NSA ≥ 2 at both 1.5 T and 3 T in this low FA region can be used for disease or response to therapy in the brain as an imaging biomarker.

The variation of the direction of the primary eigenvector of the diffusion tensor within the ROI (intra-ROI diffusion direction dispersion angle) was calculated. When SNR was high, the variation of the diffusion direction showed the uniformity of the tissue within the ROI. When the noise level of the DTI raw images was high, the measured primary diffusion direction deviated from the true direction. Therefore, we expect this variation to increase as the SNR decreases.

On the basis of this study, we suggest at least two NSAs at 3 T in the caudate nucleus are needed for determining FA on our hardware-software platforms using the ROI-based method and at least three NSAs in the putamen, thalamus lateral dorsal, ventral anterior and ventral posterior nuclei at 3 T MRI scanners.

Using the ROI-based analysis, we also suggest NSA ≥ 3 at 1.5 T and NSA ≥ 2 at 3 T in the thalamus ventral posterior nucleus are needed for determining MD and NSA ≥ 3 in the putamen, superior temporal gyrus, thalamus ventral anterior and posterior nuclei at 3 T.

Therefore, with commonly clinically acceptable NSA of 2 or 3 at 3 T, bias-free FA and MD estimation may not be obtained in many brain areas even with the ROI-based image processing offers improved reliability than the voxel-based approach. It is meaningful that among the low FA brain regions selected in this study, the method we propose is more likely to work than it is not. We have found low FA regions that work the way we propose.

In this study, the maximum DTI acquisition time is 6 min 18 sec at 3 T and a total scan time of four DTI acquisitions is greater than 25 min. DTI with NSA > 3 is not considered practical in most studies, followed by other MRI scans. However, without taking into account the patient’s DTI scan time for NSA = 4, we generated 7 groups with NSA = 4 sequentially and randomly as described in the Methods section. Our ROI-based analytical method shows that bias-free FA was obtained with NSA = 4 in all the selected regions at 3 T MRI scanners, but the bias-free FA in the CN only at 1.5 T.

As simultaneous multi-slice acquisition becomes more commonly used in diffusion imaging leading to shorter image acquisition times, bias-free estimation of FA in low FA regions of the brain could be accessible to more brain areas in the future with our proposed methodology. The FA and MD bias introduced by low SNR varies with the inherent tensor metrics of the tissue being studied, and SNR in tensor data sets should be evaluated from anatomic ROIs prior to analysis of tensor metrics.

One limitation of this study is that the method is only tested using manually defined ROIs in the native space. It is highly desirable to apply the approach to DTI normalized to a standard space such as NMI space, and define ROIs using a standard DTI atlas. However, this is beyond the scope of the current work. Another limitation of this study is that only one subject is reported, so the potential effect of variability in a population is not reflected in the study. Our work opened the opportunities for more future investigations.

The conclusions may not generalize to other vendors, protocols, MR hardware and software platform at the same vendor, parallel imaging schemes, phased array coils, magnetic field strength, gradient encoding directions, subject with abnormal or immature brain and various other technical factors. Nonetheless, in this study, the ROI-based analytic method leads to SNR enhancement and allows bias-free estimation of the DTI metrics in low FA regions of the brain while keeping the image acquisition time practical in most studies. Reliable quantification of diffusion parameters in deep brain structures with relatively low FA values may be important in the study of neurodegenerative diseases or heterogeneous disorders^6,22–29^.

## Methods

### Data acquisition

This study was approved by the institutional review board both at University of Texas Southwestern Medical Center and Korea Advanced Institute of Science and Technology with written informed consent from the participant. All investigations were carried out in accordance with relevant guidelines and regulations at our institutions. Brain DTI scans were performed on a single healthy adult volunteer (male, 40 years of age) on four different MRI scanners: an Achieva 1.5 T (Philips Healthcare System), a Discovery MR750w 3 T (GE Medical System), a Magnetom Verio 3 T (Siemens Healthcare system) and an Achieva 3 T (Philips Healthcare System). The scanner-specific DTI acquisition parameters are listed in Table 4. A total of fifteen DTI data sets were acquired using echo-planar imaging for each MRI scanner. In order to minimize head motion of the subject for a long period of scan time, three or four foam blocks were inserted between the head and the head coil to hold the head still. Each DTI data set was composed of a b = 0 image volume and 30 diffusion-weighted (DW) image volumes. Fifteen acquisitions (ACQ1, ACQ2, ACQ3, …, ACQ14 and ACQ15) were averaged to minimize bias in the very high SNR DTI data set and the FA and MD values derived from this data set were used to be the “standard reference” for each of our hardware-software MR platforms.

**Table 4:** DTI acquisition parameters on four different MRI systems.

	1.5 T Philips	3 T Philips	3 T Siemens	3 T GE
Head coil	8 channel	8 channel	12 channel	32 channel
b-values [s/mm^2^]	0^†^, 1000	0, 1000	0, 1000	0, 1000
No. of gradient directions^36^	30	30	30	30
TR [ms]	6218	8600	9700	15290
TE [ms]	100	97	93	79
FoV [mm^2^]	256 × 256	256 × 256	244 × 244	240 × 240
Acquisition matrix	128 × 128 (reconstructed to 256 × 256)	128 × 128 (reconstructed to 256 × 256)	122 × 122 (reconstructed to 256 × 256)	120 × 120 (reconstructed to 256 × 256)
Slice thickness [mm]	2	2	2	2
Voxel size [mm^3^]	(1 × 1 × 2)	(1 × 1 × 2)	(0.95 × 0.95 × 2)	(0.94 × 0.94 × 2)
No. of slices	42	40	48	52
Parallel imaging method	SENSE	SENSE	GRAPPA	ASSET
Pixel bandwidth [Hz/pixel]	1787	1742	1414	1953
Acquisition time	4 min 48 sec	5 min 12 sec	5 min 30 sec	6 min 18 sec
Total scan time	1 hour 12 min	1 hour 18 min	1 hour 22 min 30 sec	1 hour 37 min 30 sec

^†^The b = 0 images were acquired five times and averaged to enhance the SNR. In the output DTI image, there is only one b = 0 volume which is already an average of 5 acquisitions.

### Image post-processing and SNR calculation

DICOM files were exported from all MRI consoles and converted to NIFTI format using the MRIConvert tool (University of Oregon, Eugene, OR; http://old-lcni.uoregon.edu/jolinda/MRIConvert/). Data from all vendors were first inspected visually for the presence of image volumes with missing slices or large motion artifacts prior to processing. All diffusion data sets were processed offline using the FMRIB Software Library (FSL v.6.0.1)^30^. First, the brain extraction tool (BET) was used to generate a binary brain mask from the b = 0 image and remove non-brain tissue with a fractional intensity threshold of 0.3. All data were then corrected for head motion and eddy current distortions using the FSL’s EDDY tool^31,32^ by applying affine alignment of each diffusion-weighted image to the b = 0 image. All subsequent analyses were done using internally developed software written in IDL 8.4 (Exelis Visual Information Solution, Inc., Boulder, CO, USA). The SNR of DTI data sets were evaluated prior to analysis of tensor metrics. Mean signal intensity and noise were assessed from the average and subtraction of two magnitude images of consecutive acquisitions, respectively. The SNR was calculated in the average of b = 0 images as the mean voxel intensity divided by the standard deviation (SD) of voxel intensity on the subtraction image in the same ROI for a particular anatomical region for images with number of signal average (NSA) = 1–4^14^. For the standard reference image with NSA = 15, the SNR of the ROI was calculated using the SNR for image with NSA = 3 as follows^18^:

1 $$SN{R}_{NSA=15}=\surd 5\times SN{R}_{NSA=3}$$

The fifteen acquisitions were grouped separately to construct images with different NSAs sequentially and/or randomly: (i) seven tensor data sets each with NSA = 4 ((ACQ 1, 2, 3 & 4), (ACQ 5, 6, 7 & 8), (ACQ 9, 10, 11 & 12), (ACQ 12, 13, 14 & 15), (ACQ 1, 6, 10 & 15), (ACQ 2, 7, 11 & 14) and (ACQ 3, 8, 12 & 15)); (ii) eight tensor data sets each with NSA = 3 ((ACQ 1, 2 and 3), (ACQ 4, 5 and 6), (ACQ 7, 8 and 9), (ACQ 10, 11 and 12), (ACQ 13, 14 and 15), (ACQ 1, 7 and 15), (ACQ 2, 8 and 13), (ACQ 3, 9 and 14)); (iii) thirteen data sets each with NSA = 2 ((ACQ 1, 2), (ACQ 3, 4), (ACQ 5, 6), (ACQ 7, 8), (ACQ 9, 10), (ACQ 11, 12), (ACQ 13, 14), (ACQ 14, 15), (ACQ 1, 7), (ACQ 1, 15), (ACQ 8, 15), (ACQ 2, 14), (ACQ 5, 13)); and (iv) fifteen independent image sets each with NSA = 1. In construction of image sets with a specific NSA, each acquisition was used at least once.

In order to evaluate whether the potential subject motion affects SNR during a long period of scan time, the percent difference in mean SNR (%ΔSNR) between earlier ACQ groups (1, 2 and 3) and later ACQ groups (13, 14 and 15) was calculated as follows^33^:

2 $$ \% \Delta \,SNR=|\frac{Difference\,between\,two\,mean\,SNR\,values}{Average\,of\,two\,mean\,SNR\,values}|\times 100\,\,[ \% ]$$

where the mean SNR value was calculated from 10 repeated measurements in each selected brain region.

### Manual ROI-based tensor metrics quantification

Using software written in IDL 8.4, a single observer manually placed ROIs on low FA regions: head of caudate nucleus (CN), globus pallidus (GP), putamen (PUT), superior temporal gyrus (STG), and thalamus which is divided into lateral dorsal (LD), ventral anterior (VA) and ventral posterior (VP) nuclei in Supplementary Fig. 3.

For conventional quantification of tensor metrics, FA and MD are calculated for each voxel and then averaged over an ROI. This is called “voxel-based” approach. On the contrary, in an ROI-based method, the signal of the b = 0 image and all diffusion weighted images with b = 1000 s/mm^2^ was first averaged over the ROI before calculating diffusion tensor to acquire the corresponding tensor metrics. Additionally, areas from two adjacent slices were combined to form an ROI in order to further increase the ROI volume. Thus, the ROI-based approach can decrease the noise due to the spatial signal averaging and also diminish the uncertainty of the outcomes. The ROIs were drawn on the color-coded FA map where the selected regions of the brain had different colors due to distinct fiber orientations (Supplementary Fig. 3). The ROI size was calculated as follows:

3 $$ROI\,size=(numebr\,of\,voxels\,in\,the\,selected\,ROI)\times voxel\,size\,[m{m}^{3}]$$

Here the voxel refers to that of the reconstructed images, not the image acquisition voxel.

Placement of each ROI was repeated on the “standard reference” tensor data (NSA=15) until SD of the voxel-based FA was <10% for voxel-based method; the average of three FA measurements was considered representative of the voxel-based FA for each region. The last ROI was then stored and used for the constructed tensor data sets with different NSAs. For each ROI, voxel-based tensor metrics were evaluated in tensor data sets for different NSAs. In addition, for the same ROI, ROI-based tensor metrics were also obtained. From the ROI-based signals, a diffusion tensor was calculated and FA and MD were derived. ROI-based FA and MD values were compared to the conventional voxel-based ones.

### Intra-ROI diffusion direction dispersion angle (IRDDDA)

To further verify the efficacy of the ROI-based quantification method, intra-ROI diffusion direction dispersion angle (IRDDDA)^18^ was calculated to evaluate how well the diffusion directions of the voxels are aligned with each other within an ROI. Each ROI was divided into four smaller sub-ROIs. The ROI was formed across two adjacent image slices, with two sub-ROIs from each slice. First the center of the area in each slice was calculated. Then the long axis of the area was found as the line connecting any two points in the slice inside the ROI with the longest distance. The short axis of the area was defined as the line perpendicular to the long axis and passing the center of the area. The short axis divided the area into two sub-ROIs in the slice. The ROI-based diffusion tensor was calculated for all sub-ROIs and the original ROI. For each diffusion tensor, the direction of the eigenvector with the largest eigenvalue was considered as the primary diffusion direction. The angle between the primary diffusion direction of a sub-ROI and the original ROI was calculated, and the average angle for the four sub-ROIs represented the IRDDDA. When SNR is high and diffusion directions are uniform within the ROI, a small IRDDDA is expected.

### Determination of the minimum NSA for the bias-free FA and MD

If the probability of incorrectly rejecting the null hypothesis of difference between two FA values was less than 0.05, then no statistically significant difference exists. Thus, 90% confidence intervals for FA measurements were constructed (equivalence tolerance = 0.05) as follows^14,34,35^:

4 $${90}{ \% }\,confidence\,{intervals}\,of\,error=(mean\,F{A}_{i}-mean\,F{A}_{{Ref}})\pm {1.645}\times {(S{D}_{i}^{{2}}+S{D}_{{Re}f}^{{2}})}^{{0.5}}$$

where *i* is the tensor data with different NSA = 1, 2, 3 and 4; *Ref* is voxel-based value with NSA = 15; and SD is standard deviation.

If the range of the confidence interval fell entirely within the equivalence tolerance range of [−0.05, 0.05], then the measured FA was considered statistically equivalent to that derived from the standard reference data set.

Similarly, 90% confidence intervals for MD measurements were obtained as follows^19,34^:

5 $${90}{ \% }\,confidence\,intervals\,of\,error=(mean\,M{D}_{i}-mean\,M{D}_{{Ref}})\pm {1.645}\times {(S{D}_{i}^{{2}}+S{D}_{{Ref}}^{{2}})}^{{0.5}}$$

where *i* is the tensor data with different NSA = 1, 2, 3 and 4; *Ref* is voxel-based value with NSA = 15; and SD is standard deviation.

If the range of the confidence interval fell entirely within the equivalence tolerance range of [−0.05, 0.05] × 10^−3^ mm^2^/s, then the measured MD was considered statistically equivalent to the reference value.

The suggested number of acquisitions was determined in the selected regions with low FA for the NSA thresholds at four different MRI systems to avoid bias in FA and MD measurements.

## Supplementary information

Supplementary materials

## Data Availability

MRI data from this study are available to interested readers upon reasonable request.

## References

[1] Basser PJ, Pierpaoli C (1996). Microstructural and physiological features of tissues elucidated by quantitative-diffusion-tensor MRI. J Magn Reson Ser B.

[2] Le Bihan D (2001). Diffusion tensor imaging: concepts and applications. J Magn Reson Imaging.

[3] Jespersen SN, Kroenke CD, Ostergaard L, Ackerman JJ, Yablonskiy DA (2007). Modeling dendrite density from magnetic resonance diffusion measurements. NeuroImage.

[4] Mayo CD (2017). Longitudinal changes in microstructural white matter metrics in Alzheimer’s disease. NeuroImage. Clinical.

[5] Gunbey HP (2017). Structural brain alterations of Down’s syndrome in early childhood evaluation by DTI and volumetric analyses. Eur Radiol.

[6] Zhang Y (2016). Progression of Regional Microstructural Degeneration in Parkinson’s Disease: A Multicenter Diffusion Tensor Imaging Study. PloS one.

[7] Langley J (2016). Diffusion tensor imaging of the substantia nigra in Parkinson’s disease revisited. Hum Brain Mapp.

[8] Seo Y, Wang ZJ, Ball G, Rollins NK (2013). Diffusion tensor imaging metrics in neonates-a comparison of manual region-of-interest analysis vs. tract-based spatial statistics. Pediatr Radiol.

[9] Rollins NK (2010). Age-related variations in white matter anisotropy in school-age children. Pediatr Radiology.

[10] Hiratsuka Y (2007). Sensitivity of an eight-element phased array coil in 3 Tesla MR imaging: a basic analysis. Magnetic resonance in medical sciences: MRMS: an official journal of Japan Society of Magnetic Resonance in Medicine.

[11] Lin FH, Chen YJ, Belliveau JW, Wald LL (2003). A wavelet-based approximation of surface coil sensitivity profiles for correction of image intensity inhomogeneity and parallel imaging reconstruction. Hum Brain Mapp.

[12] Roemer PB, Edelstein WA, Hayes CE, Souza SP, Mueller OM (1990). The NMR phased array. Magn Reson Med.

[13] Smith SM (2006). Tract-based spatial statistics: voxelwise analysis of multi-subject diffusion data. NeuroImage.

[14] Seo Y, Wang ZJ, Morriss MC, Rollins NK (2012). Minimum SNR and acquisition for bias-free estimation of fractional anisotropy in diffusion tensor imaging - a comparison of two analytical techniques and field strengths. Magn Reson Imaging.

[15] Farrell JA (2007). Effects of signal-to-noise ratio on the accuracy and reproducibility of diffusion tensor imaging-derived fractional anisotropy, mean diffusivity, and principal eigenvector measurements at 1.5 T. J Magn Reson Imaging.

[16] Landman BA (2007). Effects of diffusion weighting schemes on the reproducibility of DTI-derived fractional anisotropy, mean diffusivity, and principal eigenvector measurements at 1.5T. NeuroImage.

[17] Wang ZJ, Seo Y, Chia JM, Rollins NK (2011). A quality assurance protocol for diffusion tensor imaging using the head phantom from American College of Radiology. Med Phys.

[18] Keller S (2018). Improvement of Reliability of Diffusion Tensor Metrics in Thigh Skeletal Muscles. Eur J Radiol.

[19] Bastin ME, Armitage PA, Marshall I (1998). A theoretical study of the effect of experimental noise on the measurement of anisotropy in diffusion imaging. Magn Reson Imaging.

[20] Pierpaoli C, Basser PJ (1996). Toward a quantitative assessment of diffusion anisotropy. Magn Reson Med.

[21] Poonawalla AH, Zhou XJ (2004). Analytical error propagation in diffusion anisotropy calculations. J Magn Reson Imaging.

[22] Pelizzari L (2019). Combined Assessment of Diffusion Parameters and Cerebral Blood Flow Within Basal Ganglia in Early Parkinson’s Disease. Front Aging Neurosci.

[23] Hori H, Yamaguchi T, Konishi Y, Taira T, Muragaki Y (2019). Correlation between fractional anisotropy changes in the targeted ventral intermediate nucleus and clinical outcome after transcranial MR-guided focused ultrasound thalamotomy for essential tremor: results of a pilot study. J Neurosurg.

[24] Harrington DL (2018). Altered Functional Interactions of Inhibition Regions in Cognitively Normal Parkinson’s Disease. Front Aging neurosci.

[25] Ramanan S (2019). Fronto-parietal contributions to episodic retrieval-evidence from neurodegenerative disorders. Learning & memory.

[26] Kaestner Erik, Reyes Anny, Macari Anna Christina, Chang Yu‐Hsuan, Paul Brianna M., Hermann Bruce P., McDonald Carrie R. (2019). Identifying the neural basis of a language‐impaired phenotype of temporal lobe epilepsy. Epilepsia.

[27] Yu, Y., Chu, L., Liu, C., Huang, M. & Wang, H. Alterations of white matter network in patients with left and right non-lesional temporal lobe epilepsy. *Eur Radiol*, 10.1007/s00330-019-06295-5 (2019). 10.1007/s00330-019-06295-5 31286187

[28] Luo C (2017). White matter microstructure damage in tremor-dominant Parkinson’s disease patients. Neuroradiology.

[29] Agosta F (2014). Mild cognitive impairment in Parkinson’s disease is associated with a distributed pattern of brain white matter damage. Hum Brain Mapp.

[30] Smith SM (2004). Advances in functional and structural MR image analysis and implementation as FSL. NeuroImage.

[31] Andersson JLR, Sotiropoulos SN (2016). An integrated approach to correction for off-resonance effects and subject movement in diffusion MR imaging. NeuroImage.

[32] Andersson JLR (2017). Towards a comprehensive framework for movement and distortion correction of diffusion MR images: Within volume movement. NeuroImage.

[33] Altman DG, Bland JM (1986). Comparison of methods of measuring blood pressure. J Epidemiol Community health.

[34] Barker LE, Luman ET, McCauley MM, Chu SY (2002). Assessing equivalence: an alternative to the use of difference tests for measuring disparities in vaccination coverage. Am J Epidemiol.

[35] Garrett KA (1997). Use of statistical tests of equivalence (bioequivalence tests) in plant pathology. Phytopathology.

[36] Jones DK, Horsfield MA, Simmons A (1999). Optimal strategies for measuring diffusion in anisotropic systems by magnetic resonance imaging. Magn Reson Med.

