# Supplementary material for: Reduction of bias in the evaluation of fractional anisotropy and mean diffusivity in magnetic resonance diffusion tensor imaging using region-of-interest methodology
Source: Sci Rep. 2019 Sep 11;9:13095. doi: 10.1038/s41598-019-49311-w (PMC6739503; doi:10.1038/s41598-019-49311-w)
Supplement: Supplementary file 1 — Supplementary materials [file 41598_2019_49311_MOESM1_ESM.pdf]

Supplementary Material

Reduction of bias in the evaluation of fractional anisotropy and mean diffusivity in magnetic resonance diffusion tensor imaging using region-of-interest methodology

Youngseob Seo, Nancy K. Rollins, Zhiyue J. Wang

Supplementary Table 1. Percent differences (%ΔSNR) in SNR between ACQ groups (1, 2 and 3) and ACQ groups (13, 14 and 15) are shown in the selected regions on 1.5 T Philips with 15 repeated DTI scan time of 72 minutes. Each SNR value was calculated from a pair of images using a difference method described in the Methods section. SNR values derived from earlier and later acquisitions differed by ≤8.1%. SNRs in earlier acquisitions are close to those in subsequent acquisitions.

Brain region		ACQ(1, 2)	ACQ(1, 3)	ACQ(2, 3)	ACQ(13, 14)	ACQ(13, 15)
CN	ACQ(1, 3)	4.9				
	ACQ(2, 3)	3.9	5.6			
	ACQ(13, 14)	6.1	6.2	5.9		
	ACQ(13, 15)	7.6	7.0	6.8	4.8	
	ACQ(14, 15)	6.8	6.4	6.1	5.7	5.5
GP	ACQ(1, 3)	5.1				
	ACQ(2, 3)	6.2	5.3			
	ACQ(13, 14)	5.5	6.5	7.3		
	ACQ(13, 15)	6.9	5.9	6.2	6.0	
	ACQ(14, 15)	6.4	7.5	7.0	5.1	6.2
PUT	ACQ(1, 3)	5.3				
	ACQ(2, 3)	4.4	5.7			
	ACQ(13, 14)	5.2	5.9	6.2		
	ACQ(13, 15)	6.5	6.1	6.3	5.4	
	ACQ(14, 15)	7.3	7.7	6.7	5.9	4.0
STG	ACQ(1, 3)	6.6				
	ACQ(2, 3)	5.7	5.9			
	ACQ(13, 14)	6.4	6.8	7.0		

	ACQ(13, 15)	6.8	6.5	6.3	5.9	
	ACQ(14, 15)	8.1	7.7	4.9	6.8	5.3
Thalamus LD	ACQ(1, 3)	3.8				
	ACQ(2, 3)	5.5	4.0			
	ACQ(13, 14)	6.4	6.9	5.8		
	ACQ(13, 15)	6.6	6.5	6.2	5.0	
	ACQ(14, 15)	7.8	6.1	6.8	4.7	5.8
Thalamus VA	ACQ(1, 3)	5.8				
	ACQ(2, 3)	4.1	6.1			
	ACQ(13, 14)	5.9	6.4	5.7		
	ACQ(13, 15)	7.5	6.3	6.9	5.1	
	ACQ(14, 15)	6.1	6.7	7.1	5.3	5.0
Thalamus VP	ACQ(1, 3)	2.4				
	ACQ(2, 3)	5.6	4.5			
	ACQ(13, 14)	6.4	5.2	5.0		
	ACQ(13, 15)	6.2	7.3	6.2	5.1	
	ACQ(14, 15)	6.7	6.9	5.5	4.7	3.6

ACQ, acquisition; CN, caudate nucleus; FA, fractional anisotropy; GP, globus pallidus; LD, lateral dorsal; MD, mean diffusivity; NSA, number of signal average; PUT, putamen; SNR, signal-to-noise ratio; STG, superior temporal gyrus; VA, ventral anterior; VP, ventral posterior.

Supplementary Table 2. Percent differences (%ΔSNR) in SNR between ACQ groups (1, 2 and 3) and ACQ groups (13, 14 and 15) are shown in the selected regions on 3 T Philips with 15 repeated DTI scan time of 78 minutes. Each SNR value was calculated from a pair of images using a difference method described in the Methods section. SNR values derived from earlier and later acquisitions differed by ≤8.3%. SNRs in earlier acquisitions are similar with those in subsequent acquisitions.

Brain region		ACQ(1, 2)	ACQ(1, 3)	ACQ(2, 3)	ACQ(13, 14)	ACQ(13, 15)
CN	ACQ(1,3)	2.9				
	ACQ(2,3)	5.0	4.7			
	ACQ(13,14)	6.0	5.1	6.1		
	ACQ(13,15)	7.2	6.9	6.1	4.4	
	ACQ(14,15)	6.9	6.4	7.8	5.7	3.8
GP	ACQ(1,3)	4.7				
	ACQ(2,3)	5.4	6.2			
	ACQ(13,14)	6.1	6.6	6.1		
	ACQ(13,15)	6.6	8.0	5.9	5.3	
	ACQ(14,15)	5.8	6.3	6.4	5.6	6.5
PUT	ACQ(1,3)	4.2				
	ACQ(2,3)	5.9	5.1			
	ACQ(13,14)	5.6	6.4	6.5		
	ACQ(13,15)	6.4	5.7	6.7	5.9	
	ACQ(14,15)	6.9	7.7	5.2	6.1	4.6
STG	ACQ(1,3)	5.8				
	ACQ(2,3)	4.5	5.6			
	ACQ(13,14)	6.3	5.9	6.6		

	ACQ(13,15)	7.9	7.5	6.0	6.0	
	ACQ(14,15)	8.3	6.5	5.3	5.7	5.9
Thalamus LD	ACQ(1,3)	4.6				
	ACQ(2,3)	5.3	5.1			
	ACQ(13,14)	5.8	5.3	6.0		
	ACQ(13,15)	6.9	6.6	6.8	5.9	
	ACQ(14,15)	7.7	6.7	5.1	4.8	4.9
Thalamus VA	ACQ(1,3)	3.9				
	ACQ(2,3)	4.7	5.1			
	ACQ(13,14)	5.4	5.1	5.7		
	ACQ(13,15)	6.2	6.9	6.6	5.8	
	ACQ(14,15)	6.1	6.3	6.7	6.1	5.1
Thalamus VP	ACQ(1,3)	1.6				
	ACQ(2,3)	4.9	3.2			
	ACQ(13,14)	6.3	5.9	6.8		
	ACQ(13,15)	6.0	7.1	6.1	5.4	
	ACQ(14,15)	7.7	6.3	6.4	6.2	4.8

ACQ, acquisition; CN, caudate nucleus; FA, fractional anisotropy; GP, globus pallidus; LD, lateral dorsal; MD, mean diffusivity; NSA, number of signal average; PUT, putamen; SNR, signal-to-noise ratio; STG, superior temporal gyrus; VA, ventral anterior; VP, ventral posterior.

Supplementary Table 3. Percent differences (%ΔSNR) in SNR between ACQ groups (1, 2 and 3) and ACQ groups (13, 14 and 15) are shown in the selected regions on 3 T Siemens with 15 repeated DTI scan time of 88 minutes. Each SNR value was calculated from a pair of images using a difference method described in the Methods section. SNR values derived from earlier acquisitions and later acquisitions differed by ≤7.9%. SNRs in earlier acquisitions are close to those in subsequent acquisitions.

Brain region		ACQ(1, 2)	ACQ(1, 3)	ACQ(2, 3)	ACQ(13, 14)	ACQ(13, 15)
CN	ACQ(1,3)	4.8				
	ACQ(2,3)	4.3	2.1			
	ACQ(13,14)	5.5	6.3	6.2		
	ACQ(13,15)	6.4	7.0	6.7	5.4	
	ACQ(14,15)	7.3	6.4	5.2	4.9	5.2
GP	ACQ(1,3)	5.3				
	ACQ(2,3)	4.2	5.7			
	ACQ(13,14)	6.4	5.9	6.8		
	ACQ(13,15)	6.7	6.9	6.8	4.6	
	ACQ(14,15)	6.3	6.4	6.6	5.5	5.7
PUT	ACQ(1,3)	3.2				
	ACQ(2,3)	5.5	5.7			
	ACQ(13,14)	6.6	6.0	7.7		
	ACQ(13,15)	6.0	6.6	5.9	5.6	
	ACQ(14,15)	6.4	6.9	6.1	6.8	4.4
STG	ACQ(1,3)	4.1				
	ACQ(2,3)	5.7	6.1			
	ACQ(13,14)	6.6	6.5	5.8		

	ACQ(13,15)	6.7	6.8	6.3	6.3	
	ACQ(14,15)	6.0	6.7	7.1	7.3	5.9
Thalamus LD	ACQ(1,3)	5.6				
	ACQ(2,3)	5.4	6.6			
	ACQ(13,14)	6.2	6.4	7.9		
	ACQ(13,15)	6.6	7.2	7.8	5.6	
	ACQ(14,15)	6.8	6.5	7.1	5.7	4.9
Thalamus VA	ACQ(1,3)	3.1				
	ACQ(2,3)	5.3	6.0			
	ACQ(13,14)	6.5	6.4	5.8		
	ACQ(13,15)	7.4	6.7	5.9	6.0	
	ACQ(14,15)	7.3	7.9	6.6	5.7	5.1
Thalamus VP	ACQ(1,3)	4.9				
	ACQ(2,3)	3.3	4.2			
	ACQ(13,14)	6.0	5.6	6.1		
	ACQ(13,15)	6.4	6.4	6.6	4.4	
	ACQ(14,15)	6.8	7.3	6.2	5.9	3.7

ACQ, acquisition; CN, caudate nucleus; FA, fractional anisotropy; GP, globus pallidus; LD, lateral dorsal; MD, mean diffusivity; NSA, number of signal average; PUT, putamen; SNR, signal-to-noise ratio; STG, superior temporal gyrus; VA, ventral anterior; VP, ventral posterior.

Supplementary Table 4. Percent differences (%ΔSNR) in SNR between ACQ groups (1, 2 and 3) and ACQ groups (13, 14 and 15) are shown in the selected regions on 3 T GE with 15 repeated DTI scan time of 97 minutes. The SNR values were calculated from a pair of images using a difference method described in the Methods section. SNR values derived from earlier acquisitions and later acquisitions differed by ≤8.2%. SNRs in earlier acquisitions are close to those in subsequent acquisitions.

Brain region		ACQ(1, 2)	ACQ(1, 3)	ACQ(2, 3)	ACQ(13, 14)	ACQ(13, 15)
CN	ACQ(1,3)	2.0				
	ACQ(2,3)	1.8	4.3			
	ACQ(13,14)	5.7	6.2	5.8		
	ACQ(13,15)	6.1	6.6	6.9	5.2	
	ACQ(14,15)	7.1	7.3	6.3	5.1	5.6
GP	ACQ(1,3)	4.3				
	ACQ(2,3)	4.9	3.8			
	ACQ(13,14)	8.0	6.5	6.3		
	ACQ(13,15)	7.6	6.5	6.9	4.9	
	ACQ(14,15)	6.5	7.3	7.0	5.1	4.7
PUT	ACQ(1,3)	4.4				
	ACQ(2,3)	3.1	4.4			
	ACQ(13,14)	5.5	6.7	6.3		
	ACQ(13,15)	6.4	7.5	6.8	6.3	
	ACQ(14,15)	7.2	6.3	7.7	6.6	4.5
STG	ACQ(1,3)	4.1				
	ACQ(2,3)	4.7	4.8			
	ACQ(13,14)	7.2	6.6	5.8		
	ACQ(13,15)	8.2	6.7	6.1	4.5	

	ACQ(14,15)	6.7	7.5	6.9	4.8	5.2
Thalamus LD	ACQ(1,3)	4.3				
	ACQ(2,3)	4.5	5.6			
	ACQ(13,14)	5.2	6.4	6.0		
	ACQ(13,15)	6.6	6.2	6.7	5.6	
	ACQ(14,15)	7.7	6.8	6.6	6.3	5.9
Thalamus VA	ACQ(1,3)	6.1				
	ACQ(2,3)	5.9	5.8			
	ACQ(13,14)	6.2	6.2	5.7		
	ACQ(13,15)	6.7	6.3	7.8	4.3	
	ACQ(14,15)	7.1	8.1	6.5	5.8	4.1
Thalamus VP	ACQ(1,3)	3.8				
	ACQ(2,3)	2.9	3.5			
	ACQ(13,14)	6.7	5.1	7.5		
	ACQ(13,15)	5.8	6.9	6.4	5.2	
	ACQ(14,15)	6.6	7.5	7.0	5.8	6.4

ACQ, acquisition; CN, caudate nucleus; FA, fractional anisotropy; GP, globus pallidus; LD, lateral dorsal; MD, mean diffusivity; NSA, number of signal average; PUT, putamen; SNR, signal-to-noise ratio; STG, superior temporal gyrus; VA, ventral anterior; VP, ventral posterior.

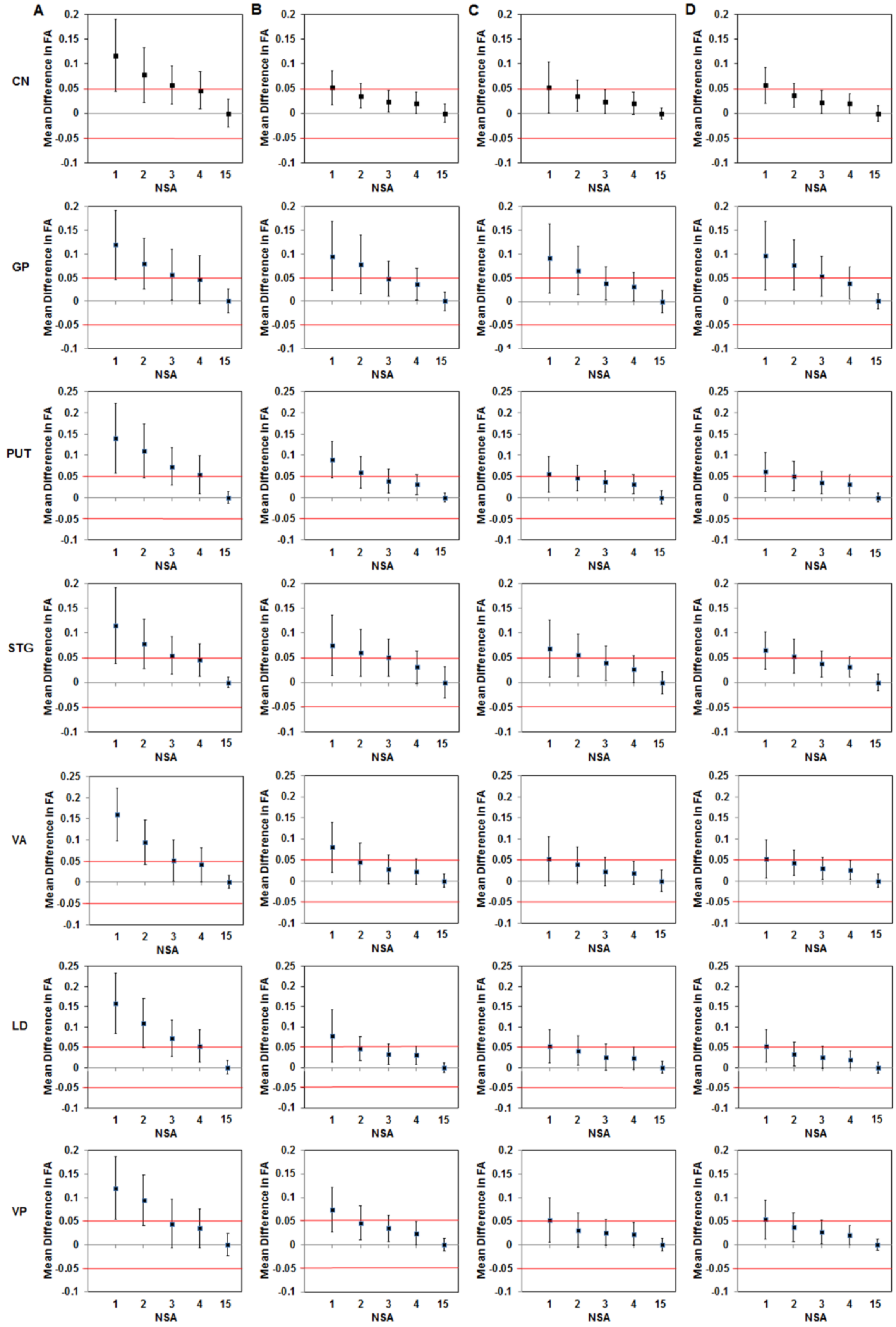
A
B
C
D
CN
GP
PUT
STG
VA
LD
VP
Mean Difference in FA
NSA
1
2
3
4
15

Supplementary Figure 1. Equivalence testing for the determination of the minimum NSA for bias-free FA evaluation is shown at voxel-based analysis at 1.5 T Philips (A), 3 T Philips (B), 3 T Siemens (C) and 3 T GE (D) MRI systems (Equivalence tolerance range = [-.05, .05]). FA, fractional anisotropy; NSA, number of signal average. While the 90% CIs of voxel-based FA at 1.5 T Philips were not within the equivalence tolerance range for all the selected regions, for voxel-based FA at 3 T Philips, Siemens and GE, CN only had equivalence threshold at NSA=3, and CN, VA, LD and VP had equivalence threshold at NSA=4.

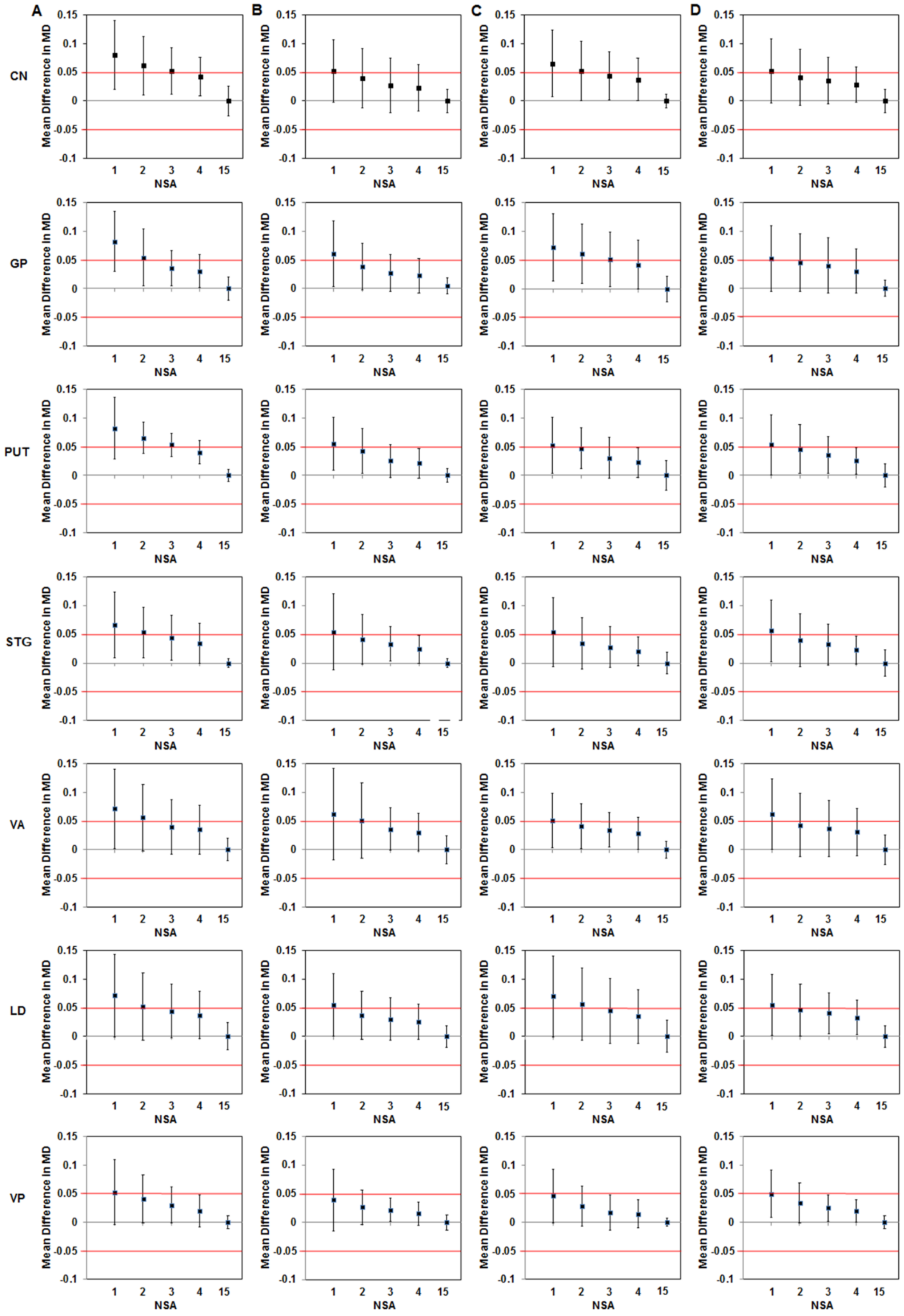
A
B
C
D
CN
GP
PUT
STG
VA
LD
VP
Mean Difference in MD
NSA
0.15
0.1
0.05
0
-0.05
-0.1
1
2
3
4
15

Supplementary Figure 2. Equivalence testing for the determination of the minimum NSA for bias-free MD evaluation is shown at voxel-based analysis at 1.5 T Philips (A), 3 T Philips (B), 3 T Siemens (C) and 3 T GE (D) MRI systems (Equivalence tolerance range = [-.05, .05]). MD, mean diffusivity; NSA, number of signal average. For voxel-based MD at 1.5 T Philips, CIs were not within the equivalence tolerance range of [-0.05, 0.05] for all the selected regions of the brain except VP, while for voxel-based MD at 3 T Philips, Siemens and GE, PUT and STG had equivalence threshold at NSA=4 and VP at NSA≥3.

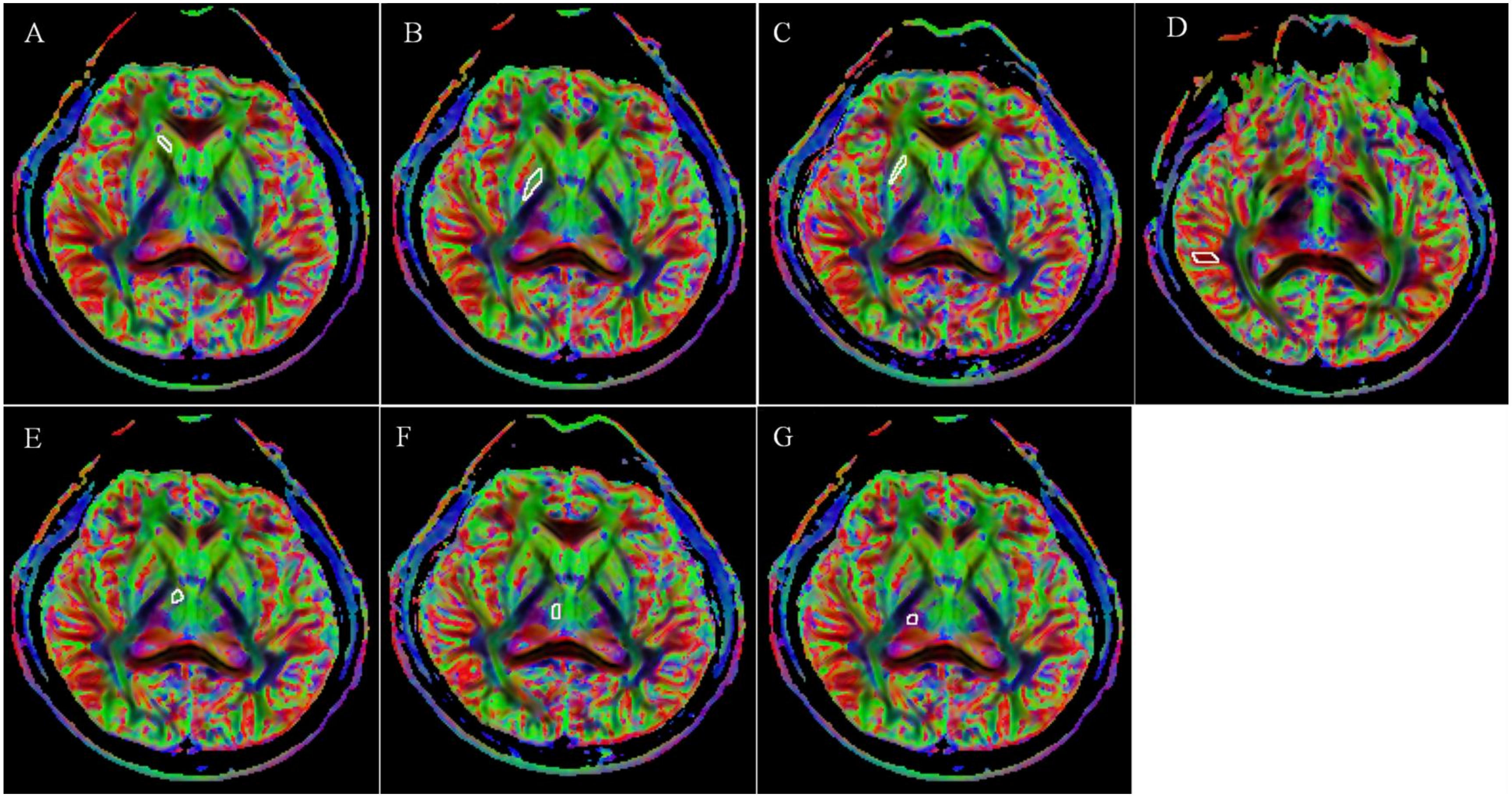

Supplementary Figure 3. ROIs (white) of CN (A), GP (B), PUT (C), STG (D), VA (E), LD (F) and VP (G) are placed for ROI-based and voxel-based analysis. The primary diffusion direction was color-coded but modulated by (1-FA) value to allow better visualization of low FA areas. CN, caudate nucleus; GP, globus pallidus; LD, lateral dorsal; NSA, number of signal average; PUT, putamen; ROI, region-of-interest; SNR, signal-to-noise ratio; STG, superior temporal gyrus; VA, ventral anterior; VP, ventral posterior.
